# Nrf2-ARE Signaling Partially Attenuates Lipopolysaccharide-Induced Mammary Lesions via Regulation of Oxidative and Organelle Stresses but Not Inflammatory Response in Mice

**DOI:** 10.1155/2021/8821833

**Published:** 2021-01-08

**Authors:** Yongxin Li, Juanjuan Shao, Pengfei Hou, Feng-Qi Zhao, Hongyun Liu

**Affiliations:** ^1^College of Animal Sciences, Zhejiang University, Hangzhou 310058, China; ^2^Department of Animal and Veterinary Sciences, University of Vermont, Burlington, VT 05405, USA

## Abstract

The incidence of mastitis is high during the postpartum stage, which causes severe pain and hinders breast feeding in humans and reduces milk production in dairy cows. Studies suggested that inflammation in multiple organs is associated with oxidative stress and nuclear factor E2-related factor 2 (Nrf2)-antioxidant response element pathway is one of the most important antioxidant pathways, but the effects of Nrf2 on antioxidation in the mammary gland during mastitis are still unclear. In this study, intramammary lipopolysaccharide (LPS) challenge was carried out in wild-type (WT) and Nrf2 knockout mice. Results showed that the expression of Nrf2 affected the expression of milk protein genes (Csn2 and Csn3). Importantly, LPS treatment increased the expression of Nrf2 and HO-1 and the content of glutathione in the mammary gland of WT mice, but not in Nrf2(-/-) mice. The expression levels of glutathione synthesis genes (GCLC, GCLM, and xCT) were lower in Nrf2(-/-) mice than in WT mice. Moreover, mitochondrial-dependent apoptotic and endoplasmic reticulum stress were significantly relieved in WT mice compared with that in Nrf2(-/-) mice. In summary, the expression of Nrf2 may play an important role in prevention of oxidative and organelle stresses during endotoxin-induced mastitis in mouse mammary gland.

## 1. Introduction

Milk is important for the growth and development of the youth and nutrition of humans. Unfortunately, the incidence of mastitis arises during lactation, which causes severe pain and even sepsis and hinders breast feeding in humans and reduces milk production in dairy cows [1, 2]. Gram-negative bacteria *Escherichia coli* is one of the main pathogens responsible for mastitis, and lipopolysaccharide (LPS) is a major component of the outer cell membranes of gram-negative bacteria. The mammary gland is highly sensitive to LPS, and LPS-induced model is a valuable tool to study the coliform mastitis [2].

Studies have shown that injury in several organs are closely related to oxidative stress and inflammation, such as kidney injury and liver injury [3, 4], and the production of reactive oxygen species is considered central to the progression of some inflammatory diseases [5]. Nuclear factor E2-related factor 2 (Nrf2)-antioxidant response element (ARE) pathway played a key role in antioxidation in the body and was found essential for cytoprotective effects of astragaloside IV in epithelial cells of mammary glands [6]. Therefore, the role of inflammation and oxidative stress in mammary lesions is worth studying.

Nrf2-ARE signaling pathway is activated when oxidative stress occurs and promotes the expression of multiple endogenous antioxidants and phase II detoxification enzymes [7]. Some research explored the target genes of Nrf2-ARE pathway through gene chip systematically and found that it could regulate the expression of more than 200 genes [8]. However, whether the deficiency of Nrf2 has a negative effect on mastitis and oxidative stress in the mammary gland has not been studied. The aim of this study was to explore the effects of Nrf2-ARE pathway in the mastitis using wild-type (WT) and Nrf2 knockout mice with a self-controlled experimental design.

## 2. Materials and Methods

### 2.1. Animals

Nrf2 knockout mice (B6.129X1-Nfe2l2^tm1Ywk^/J) were purchased from the Jackson Laboratory (Bar Harbor, ME, USA) [9], and WT mice were purchased from Laboratory Animal Center of Zhejiang University (Hangzhou, China). Nrf2(+/-) mice were obtained by mating of Nrf2(-/-) mice with WT mice, and self-crossing of the Nrf2(+/-) mice produced F2 offspring of WT mice, Nrf2(+/-) mice, and Nrf2(-/-) mice [10]. These mice were housed individually and kept under a 12 h light/dark photoperiod (lights on at 09:00, lights off at 21:00) under controlled temperature (24°C ± 1°C) and humidity (55% ± 5%). Food and water were available ad libitum. All experimental procedures and protocols were approved by the Institutional Animal Care and Use Committee in Zhejiang University. Female mice in early and middle lactation (day 3 and day 12) at 10-12 weeks of age were used for the subsequent study.

### 2.2. Experimental Treatments

Fifteen female mice (*n* = 15) were used in each of the following groups: (a) WT mice (control group), (b) Nrf2(+/-) mice, and (c) Nrf2(-/-) mice. The pups were removed from the mothers 1 h before the LPS injection. For intramammary injection, the right and left sides of 4^th^ mammary glands (L4 and R4) of every mouse were alternatively injected through the teat meatus with 50 *μ*l LPS (Sigma Chemical Co, St. Louis, USA; 10 *μ*g LPS from *Escherichia coli* O111:B4 dissolved in 50 *μ*l PBS) or 50 *μ*l PBS, respectively. The two glands were thoroughly and gently massaged after injection [11]. At 3 h after injection, milk was collected from the midlactation mice (day 12) with injection of oxytocin [12]. At 24 h after injection, the mice of early lactation (day 3) were sacrificed, and the R4 and L4 mammary glands were individually collected.

### 2.3. Genotyping

DNA was extracted from mouse tissue samples of toes and mammary gland using the Rapid Genotype Identification Kit (Beyotime, Shanghai, China). Mice were genotyped by PCR using the procedures provided by the Jackson Laboratory [9] (Supplemental Figure 1).

### 2.4. Feed Intake, Body Weight, and Reproductive Performance

The maternal mice and leftover feed were weighed at 10:00 every day before mammary injection, and feed intake was calculated by the 24 h feed consumption. Litter size was recorded, and litter weight was weighed on the third day of lactation. Litter weight per pup was calculated by litter weight divided by litter size.

### 2.5. Milk Production and Milk Component Analysis

On the 6th day to 11th day of lactation, litter weight (W1) was measured at 10:00, and the pups were isolated from their mother for 4 h; then, litter weight (W2) was measured at 14:00. After that, the pups and their mother were placed in a cage for 2 h; then, litter weight (W3) was measured at 16:00. Calculation formula of milk production was as follows: milk production = (W3 − W2) + (W1 − W2)/2 [12].

The concentration of milk protein was determined using the BCA Assay Kit (Beyotime). The triglyceride concentration was measured using the Triglyceride Colorimetric Assay Kit (Cayman Chemical, Ann Arbor, USA), and lactose concentration was measured using the Lactose Assay Kit (Cell Biolabs, San Diego, USA).

### 2.6. Hematoxylin-Eosin (H&E) Staining

Small pieces of mammary tissues were fixed in 4% paraformaldehyde and dehydrated in ethanol. After paraffin embedding, 5 *μ*m sections were cut and stained with H&E. H&E-stained sections were observed at a magnification of 200 under a light microscope. Three visual fields were captured for each slice with an optical microscope with a digital camera (ECLIPSE 80i, Nikon, Japan). Image Pro Plus 6.0 (Media Cybernetics, Silver Spring, MD, USA) was used for H&E-stained sections analysis. The degree of infiltration of inflammatory cells was calibrated by the area of mammary gland alveoli.

### 2.7. RNA Isolation and Quantitative Real-Time PCR (qPCR)

Total RNA was extracted from the mammary tissues using the RNA Pure Kit according to the manufacturer's procedures (Aidlab Biotechnologies Co., Ltd, Beijing, China). Total RNA of 1000 ng was reverse transcribed to cDNA using the PrimeScript RT reagent (Takara, Tokyo, Japan). Primers for qPCR were designed using the National Center for Biotechnology Information Primer-BLAST site and listed in Supplemental Table 1. qPCR was performed in a 7500c real-time PCR detection system (Applied Biosystems, Carlsbad, California, USA) using SYBR premix EX Taq (Takara). SDHA, HPRT1, and ARBP were selected as reference genes from six housekeeping genes (SDHA, HPRT1, ARBP, GAPDH, *β*-actin, and B2M) using geNorm [13, 14]. The 2^−ΔΔCt^ method was used to calculate the relative mRNA expression of genes.

### 2.8. RNA Sequencing

Mammary gland tissues from 4 animals in WT and Nrf2(-/-) groups were randomly selected for RNA sequencing (*n* = 4). TRIzol reagent was used to extract total RNA from each sample (Invitrogen, CA, USA). RNA integrity was assessed with RNA Nano 6000 Analysis Kit in the Bioanalyzer 2100 System (Agilent Technologies, CA, USA). In total, 16 samples were qualified to transcriptome analysis. A total amount of 3 *μ*g RNA per sample was used as input material for the RNA sequencing library preparations. The libraries were sequenced by the high-throughput Illumina sequencing platform (HiSeq 4000) of Novogene Bioinformatics Institute (Beijing, China) using standard procedures (https://www.novogene.com/tech/service/etwithref/product/).

### 2.9. Bioinformatic Analysis

After removing reads containing adapter or ploy-N and low quality (Phred score *Q* ≤ 20) reads, clean reads were generated. The fragments per kilobase million of each gene were calculated based on the length of the gene. The fold change of each differentially expressed gene (DEG) was calculated. DEGs were identified based on Benjamini and Hochberg's method by calculating the *P*-adjust (*P*_adj_ < 0.01) and fold change (∣log_2_FC | >1) [15]. DEGs were analyzed for Gene Ontology (GO) enrichment and Kyoto Encyclopedia of Genes and Genomes (KEGG) pathway analysis. Interaction networks of DEGs were obtained using the STRING v10.5 database (http://string-db.org/). Furthermore, hub genes were determined using Cytoscape_v3.6.0 and subnets were selected by Mcode 1.5.1 in Cytoscape_v3.6.0. GO analysis was used for DEGs in these subnets to explore functional enrichment [12].

### 2.10. Measurement of Total Antioxidant Capacity (T-AOC) and Glutathione (GSH)/Oxidized Glutathione (GSSG)

The T-AOC was determined by the Total Antioxidant Capacity Assay Kit with ABTS method (Beyotime), and calibrated by protein concentration which was determined using BCA Assay Kit (Beyotime). The levels of GSH and GSSG were measured by GSH and GSSG Assay Kit (Beyotime) and calibrated by the weight of tissues, according to the kit's instructions (*n* = 6).

### 2.11. Statistical Analysis

Data are presented as mean ± SEM. Paired *t* test or ANOVA followed by Tukey's multiple comparison were used for data analysis using IBM SPSS Statistics 19 (IBM, Armonk, NY, USA) to find the differences between LPS and PBS treatments, or among three genotypes of mice. *P* < 0.05 was considered statistically significant. GraphPad Prism Software version 6.0 (GraphPad Software Inc., La Jolla, CA, USA) was used for graphing.

## 3. Results

### 3.1. Lactation Performance and Expression of Milk Protein Genes

**Figure 1:**
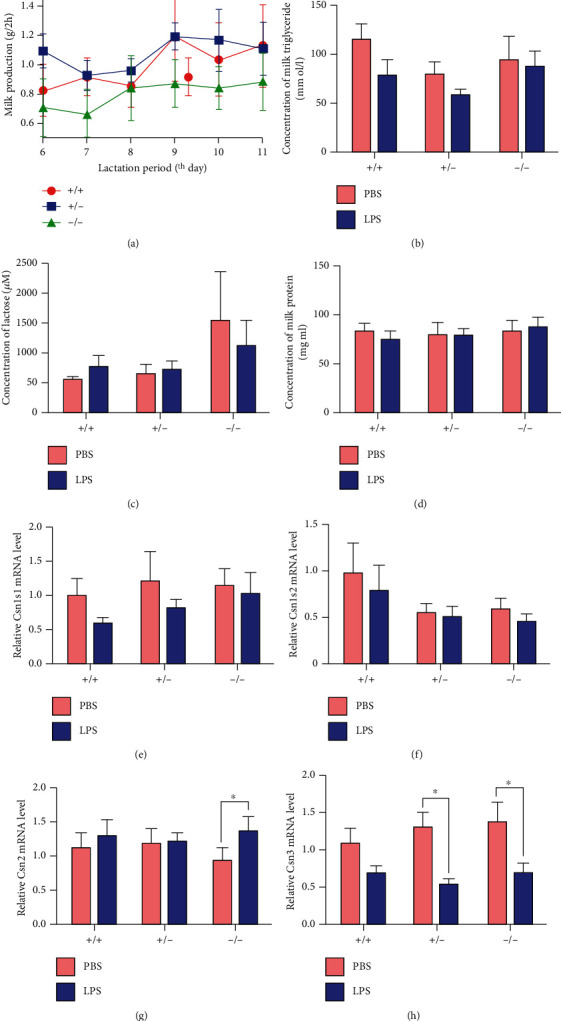
Effects of Nrf2 genotype on lactation performance and expression of milk protein genes in mice. Effects of Nrf2 genotype on (a) milk production on the 6th day to 11th day of lactation (*n* = 5), the concentrations of (b) milk triglyceride, (c) lactose, and (d) milk protein on day 12 in lactation (*n* = 5), and relative mRNA abundance of (e) Csn1s1, (f) Csn1s2, (g) Csn2, and (h) Csn3 in the mammary glands of WT, Nrf2(+/-), and Nrf2(-/-) mice treated with either LPS or PBS (*n* = 6). In all panels, *t* test was used to determine the differences between LPS and PBS treatments. ANOVA followed by Tukey's multiple comparison was used to determine the differences among three genotypes of mice. Data represent mean ± SEM. All data marked with ^∗^ represent a significant difference (*P* < 0.05).

The expression of Nrf2 did not affect body weight and feed intake among three genotypes of mice (Supplemental Figure 1). On the 6th day to 11th day of lactation, the expression of Nrf2 did not affect milk yield of maternal mice significantly (Figure 1(a)). LPS challenge did not affect milk protein, milk fat, and lactose content on day 12 in lactation (Figures 1(b)–1(d)), but reduced the expression of Csn3 in Nrf2(-/-) and Nrf2(+/-) mice and showed a decreasing trend in WT mice (Figure 1(h)). Meanwhile, LPS treatment significantly increased the expression of Csn2 in Nrf2(-/-) mice but had no effect on the expression of Csn1s1 and Csn1s2 in all groups (Figures 1(e)–1(g)).

### 3.2. Morphological Changes and Inflammatory Cytokines

**Figure 2:**
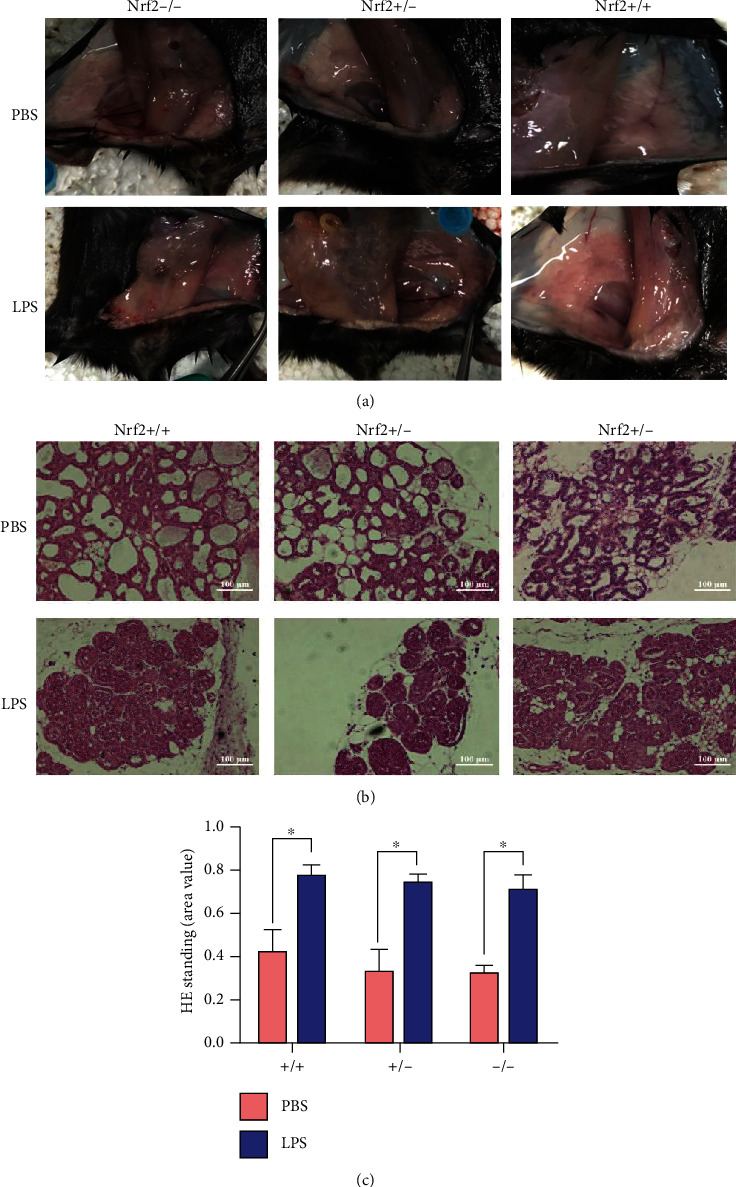
Effects of LPS and Nrf2 on morphological changes and cell infiltration of the mammary gland in WT, Nrf2(+/-), and Nrf2(-/-) mice. (a) Morphological change, (b) H&E staining, and (c) statistical result of H&E staining. In (c), *t* test was used to determine the differences between LPS and PBS treatments. Data represent mean ± SEM (*n* = 3), and data marked with ^∗^ represent a significant difference (*P* < 0.05).

**Figure 3:**
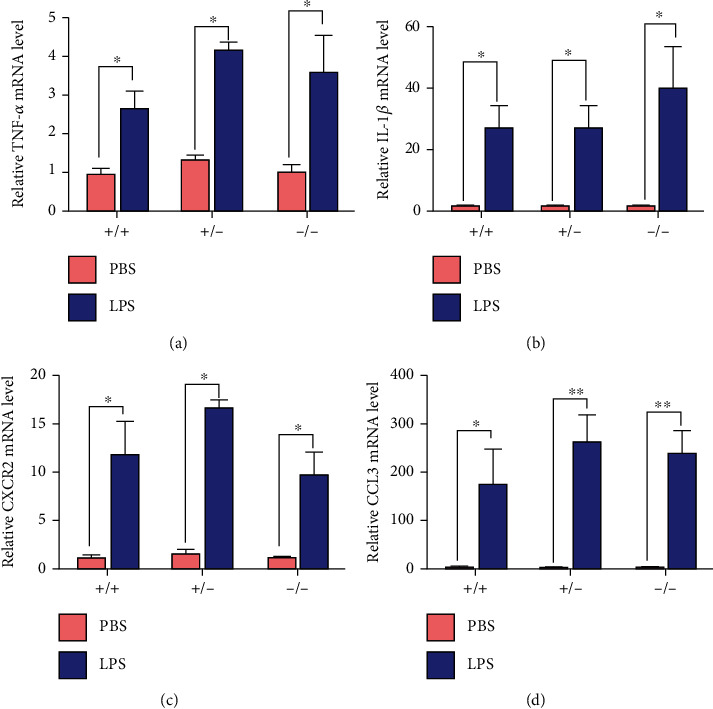
Effects of LPS and Nrf2 genotype on inflammatory cytokines. Effects of LPS and Nrf2 genotype (+/+, +/-, and -/-) on relative mRNA abundance of (a) TNF-*α*, (b) IL-1*β*, (c) CXCR2, and (d) CCL3 in the mammary glands. In all panels, *t* test was used to determine the differences between LPS and PBS treatments. Data represent mean ± SEM (*n* = 6). All data marked with ^∗^ represent a significant difference (0.01 < *P* < 0.05), and marked with ^∗∗^ represent a highly significant difference (*P* < 0.01).

LPS treatment induced inflammation in the mammary gland in all three mice, demonstrated by the red appearance (Figure 2(a)). The H&E-staining showed that LPS injection induced the infiltration of inflammatory cells in the mammary gland of all mice (Figure 2(b)), and the degree of infiltration of inflammatory cells did not show significant difference among the genotypes (Figure 2(c)). Meanwhile, LPS stimulation promoted the mRNA abundance of inflammatory cytokines (TNF-*α* and IL-1*β*), chemokines (CCL3), and chemokine receptors (CXCR2) in the mammary gland of all mice after 24 h (Figures 3(a)–3(d)).

### 3.3. GO and KEGG Enrichment Analysis of RNA Sequencing

After calculating the fragments per kilobase million of all genes in each sample, the box plot showed the same treatment of different genotype mice had a similar distribution of gene expression (Supplemental Figure 2). The qPCR results (Supplemental Figure 3) showed that nearly 70% of the changes in gene expression were consistent with the results of RNA-seq analysis. In total, 476 and 476 GO terms and 24 and 28 KEGG terms were significantly (*P*_adj_ < 0.05) enriched in DEGs between LPS and PBS treatments in WT mice and in Nrf2(-/-) mice, respectively. The functions of DEGs were mainly enriched in single cell adhesion, chemotaxis, leukocyte migration, leukocyte-cell adhesion, T cell activation, and cytokine-mediated signaling pathways in both WT and Nrf2(-/-) mice (Supplemental Figure 4). For KEGG enrichment analysis, the DEGs of WT and Nrf2(-/-) mice are enriched in cytokine and cytokine receptor interactions, Toll-like receptor signaling pathway, NF-*κ*B signaling pathway, IL-17 signaling pathway, NOD-like receptors signaling pathway, TNF signaling pathway, chemokine signaling pathway, B cell receptor signaling pathway, and so on (Supplemental Figure 4).

### 3.4. Protein-Protein Interaction (PPI) Network Analysis

**Figure 4:**
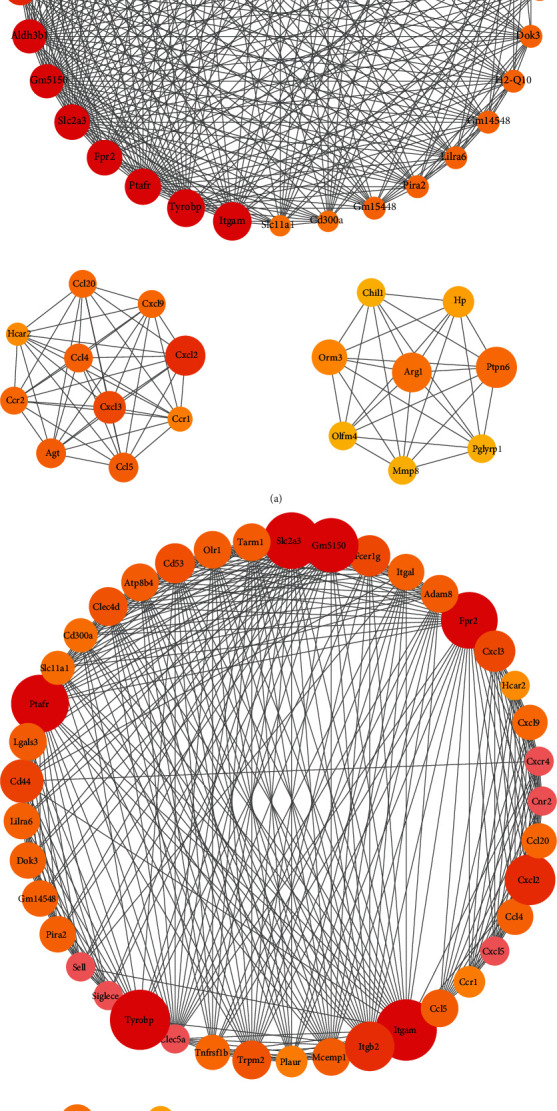
Protein-protein interaction (PPI) network analysis in WT and Nrf2(-/-) mice, respectively (*n* = 4). (a) Summary of PPI subnets in WT mice between LPS and PBS treatments. (b) Summary of PPI subnets in Nrf2(-/-) mice between LPS and PBS treatments.

A total of 199 and 187 nodes as well as 845 and 867 edges were included in the PPI network in WT and Nrf2(-/-) mice, respectively (Supplemental Figure 5). CD53 and Clec4d were at the core of the network in WT mice. C5ar1 and Ccl5 occupied the key regulatory position in the network of Nrf2(-/-) mice. The functional enrichment of DEGs in key subnets was explored (Figures 4(a) and 4(b)). There were many inflammation-related subnets in the two genotypes of mice, and their main functions were enriched in leukocyte migration, immune response regulation, and cell adhesion.

### 3.5. Nrf2-ARE Antioxidant Pathway and GSH Metabolism

**Figure 5:**
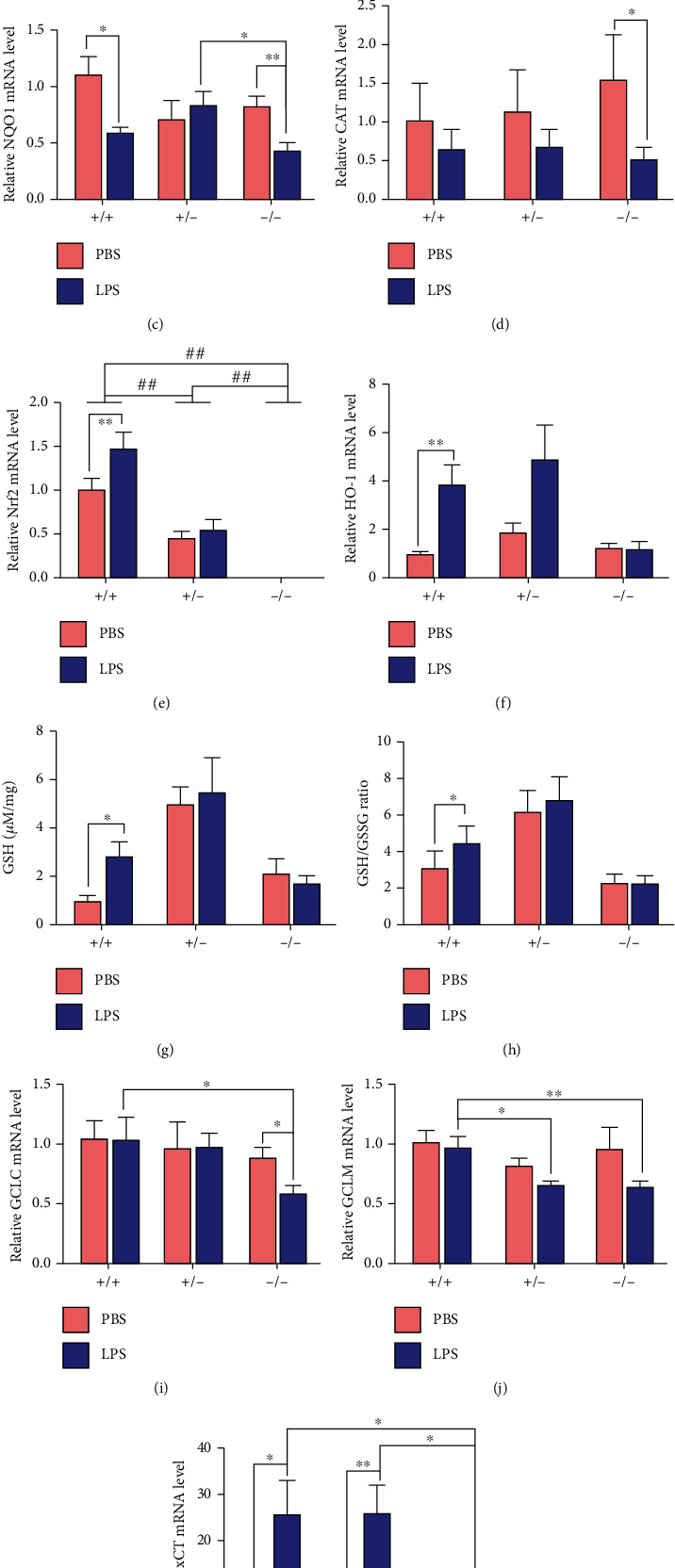
Effects of LPS and Nrf2 phenotype (+/+, +/-, and -/-) on oxidative stress and antioxidation in the mammary gland. (a) mRNA expression changes in genes of antioxidation in the LPS treatment groups between WT and Nrf2 (-/-) mice. Data represents the log_2_(foldchange) (*n* = 4). Data marked with ^∗∗^ represent 0.001 < *P*_adj_ < 0.01, and marked with ^∗∗∗^ represent *P*_adj_ < 0.001. (b–k): (b) T-AOC, the protein abundance of (g) GSH, (h) GSH/GSSG ratio, mRNA abundance of (c) NQO1, (d) CAT, (e) Nrf2, (f) HO-1, (i) GCLC, (j) GCLM, and (k) xCT in mice. *T* test was used to determine the differences between LPS and PBS treatments. ANOVA followed by Tukey's multiple comparison was used to determine the differences among three genotypes of mice. Data represent mean ± SEM (*n* = 6). The data marked with ^∗^ represent a significant difference (0.01 < *P* < 0.05), and marked with ^∗∗^ or ^##^ represent a highly significant difference (*P* < 0.01).

RNA-seq showed that the mRNA levels of several Nrf2-traget antioxidant genes (HO-1, xCT, and GCLM) were significantly decreased in Nrf2(-/-) mice compared with WT mice after LPS treatment (Figure 5(a)). LPS challenge reduced the expression of NQO1 in both WT and Nrf2(-/-) mice, reduced T-AOC, and inhibited the expression of the antioxidant enzymes CAT in Nrf2(-/-) mice but not in WT mice (Figures 5(b)–5(d)). Meanwhile, LPS increased the expression of Nrf2 and its downstream gene HO-1 significantly in WT mice, while these changes did not occur in Nrf2(-/-) mice (Figures 5(e) and 5(f)). The GSH content and GSH/GSSG ratio were significantly increased after LPS challenge in WT mice, whereas there was no difference in Nrf2(+/-) and Nrf2(-/-) mice (Figure 5(g) and 5(h)). Furthermore, the expression of GSH synthesis genes (GCLC, GCLM, and xCT) was lower by LPS challenge in Nrf2(-/-) mice compared with in WT mice (Figures 5(i)–5(k)).

### 3.6. Mitochondrial-Dependent Apoptotic and Endoplasmic Reticulum (ER) Stress

**Figure 6:**
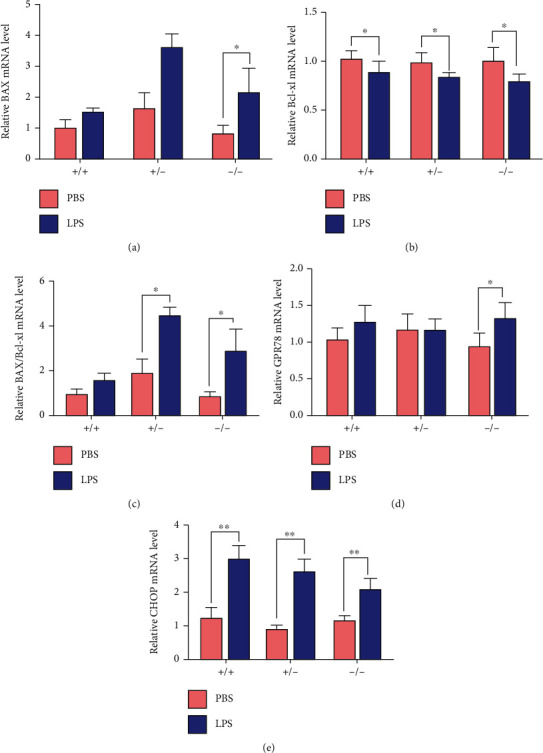
Effects of LPS and Nrf2 phenotype on mitochondrial-dependent apoptotic and endoplasmic reticulum stress. Effects of LPS and Nrf2 phenotype (+/+, +/-, and -/-) on relative mRNA abundance of (a) BAX, (b) Bcl-xl, (c) BAX/Bcl-xl, (d) GPR78, and (e) CHOP in the mammary gland of mice. In all panels, *t* test was used to determine the differences between LPS and PBS treatments. Data represent the mean ± SEM (*n* = 6). All data marked with ^∗^ or ^#^ represent a significant difference (0.01 < *P* < 0.05), and marked with ^∗∗^ or ^##^ represent a highly significant difference (*P* < 0.01).

LPS treatment increased the expression of proapoptotic factor (BAX) in Nrf2(-/-) mice and reduced the expression of antiapoptosis factor (Bcl-xl) in all mice (Figures 6(a) and 6(b)). Moreover, the BAX/Bcl-xl ratio was increased in Nrf2 knockout mice (Figure 6(c)). LPS treatment increased the expression of ER stress markers (GPR78 in Nrf2(-/-) mice and CHOP in all mice) (Figures 6(d) and 6(e)).

## 4. Discussion

The incidence of mastitis is high during postpartum lactation, which results in reduced breast feeding in humans and milk production in dairy animals [1, 2]. Studies have shown that inflammation in multiple organs was associated with oxidative stress [5], but it has not been explored clearly in the mammary gland. In this study, we successfully adapted the LPS challenge model in the mammary gland of lactating mice to explore the role of Nrf2 on mastitis and oxidative stress.

Research has found that subclinical mastitis affected protein composition of milk [16], and LPS-induced mastitis affected the expression of milk protein genes (Csn1s1 and Csn2) [17]. Therefore, we investigated whether LPS challenge and Nrf2 genotype affected milk composition. Results showed that Nrf2 had no significant effect on milk yield and milk protein content which probably due to the simultaneous up-regulation of Csn2 and down-regulation of Csn3.

In our study, the H&E-staining and expression of inflammatory cytokines showed that intramammary LPS treatment for 24 hours resulted in mammary inflammation, which was consistent with previous study [2, 18]. The expression of Nrf2 did not affect the degree of inflammatory cell infiltration and secretion of inflammatory factors, demonstrating that Nrf2 may not play a role in the process of mammary inflammation obviously.

Subsequently, transcriptomics was analyzed to observe the effects of LPS treatment on genes, physiological processes, and signaling pathways in WT and Nrf2(-/-) mice. Results showed that 25 and 27 DEGs were enriched in cytokine-cytokine receptor interactions in WT and Nrf2(-/-) mice, respectively. CCL4, CCL5, and CXCL9 are all chemokine ligands [19], which were significantly up-regulated, suggesting that chemokines participated in LPS-induced inflammation. We also found that 9 and 11 DEGs were enriched in the Toll-like receptor pathway in WT mice and Nrf2(-/-) mice, respectively, including IL1*β*, TNF, and IKBKE. Toll-like receptors are known receptor of LPS, which activate the downstream signaling pathways, recruited large numbers of neutrophils and phagocytic cells locally, and released proinflammatory cytokines to stimulate innate immune response [20]. The results revealed that these inflammatory factors played an important role in LPS-induced mammary inflammation in both WT and Nrf2(-/-) mice.

Another inflammation-related pathway enriched in LPS treatment animals was NF-*κ*B signaling pathway. Previous studies showed that anti-inflammatory biochemicals inhibited NF-*κ*B and activated Nrf2-ARE signaling pathway simultaneously, suggesting that NF-*κ*B and Nrf2 pathways synergistically regulated the anti-inflammatory function [21, 22]. However, our study showed no difference in NF-*κ*B signaling between two Nrf2 genotypes and suggested that Nrf2 may be not involved in LPS activated NF-*κ*B pathways in the mammary gland.

C-type lectin domain family accounted for a large proportion in hub genes of PPI network. The family of proteins is an important component of pattern recognition receptor and has been shown to be necessary in mediating immune and inflammation and defending against fungal infections [23, 24]. Members of this family are involved in multiple processes of cell adhesion, cell-cell signaling, glycoprotein conversion, and immune response [23]. C-type lectin domain family 4 member D (Clec4d) was a hub gene found in both WT and Nrf2(-/-) mice, with the degree of 21, and its expression was significantly increased after LPS treatment. Clec4d may trigger intracellular signaling and induce phagocytosis and the release of proinflammatory cytokines [25, 26]. A hub gene in Nrf2(-/-) mice is C-type lectin domain family 5 member A (Clec5a) which has been found to be involved in the progression of a variety of chronic inflammatory diseases [27]. For example, Clec5a acted as a signaling receptor for proinflammatory cytokine release in dengue virus infection [28]. Our results revealed C-type lectin domain family was tightly involved in the pathogenesis of LPS reduced mammary lesions.

RNA-seq showed that several genes (HO-1, xCT, and GCLM) closely related to antioxidation were down-regulated in LPS-treated group of Nrf2(-/-) mice, compared with that in WT mice. To study the possible mechanism of LPS-induced oxidative stress in the mammary gland, the expression changes of Nrf2 and its downstream genes after LPS challenge were studied. LPS treatment raised the expression of Nrf2 in WT mice and decreased T-AOC and antioxidant enzymes expression in Nrf2(-/-) mice, suggesting that Nrf2-ARE may play a role in antioxidant function in the mammary gland. Moreover, LPS treatment up-regulated the expression of HO-1 in WT mice, but not in Nrf2(-/-) mice, indicating that the expression of HO-1 was highly dependent on the presence and activation of Nrf2, which was consistent with other findings [29, 30].

Many studies found that Nrf2 is activated when the tissue is exposed to stress with increased GSH level and GSH/GSSG ratio to resist stress injury [31]. For example, Nrf2 mediated the increased synthesis of GSH in stressed astrocytes [32]. There were studies showed that glutamate cysteine ligase (GCL) was the rate-limiting enzyme in the synthesis of GSH, and the cystine antiporter-xCT transported the precursors of GSH synthesis. Nrf2 may influence the modulatory subunit and catalytic subunit (GCLM, GCLC) of GCL and the cystine antiporter xCT, to affect the level of GSH [33, 34]. However, there was also a report that the increased expression of GCLC and xCT was independent on Nrf2 in colorectal carcinoma cells [35]. In our results, LPS treatment promoted the expression of xCT and increased the GSH content and GSH/GSSG ratio as remedial measures to resist oxidative stress in WT mice. In Nrf2(-/-) mice, the up-regulation of xCT was less than that in WT mice. In addition, Nrf2(-/-) mice could not maintain the normal level of the subunits of GCL (GCLC, GCLM) as shown in WT mice, leading to the weakness of oxidation resistance.

Wu et al. [36] reported LPS treatment activated apoptosis-related pathways in liver, and Nrf2 was activated during the induction of ER stress in human [37]; therefore, we speculated that LPS stimulation may cause mammary tissue damage through apoptosis and organelle injury, which may be protected by Nrf2. Indeed, in the present study, LPS treatment increased the expression of BAX and BAX/Bcl-xl ratio in Nrf2(-/-) mice compared to WT mice, suggesting that Nrf2 may influence the mitochondrial-dependent apoptosis. These results were consistent with previous report that Nrf2 deficiency aggravated apoptosis in inflammation-associated lung injury [38].

## 5. Conclusion

In summary, Nrf2(-/-) mice were subjected to more severe oxidative stress and organelle stress after LPS challenge, and the protective effects of Nrf2-ARE may be partly due to Nrf2/GCL/GSH pathway, but the activation of Nrf2 was not sufficient to resist inflammatory injury. This study innovatively revealed the relationship between Nrf2-ARE pathway and mammary injury, which provides a theoretical basis for the research of mammary lesions.

## Data Availability

The raw sequence data were deposited in the NCBI Sequence Read Archive. The reviewer's link of the raw sequence data is as follows: https://dataview.ncbi.nlm.nih.gov/object/PRJNA631134?reviewer=pq0nvmjivcsum0p5ph7etkhlrl The data used to support the findings of this study are available from the corresponding author upon request.

## Abbreviations

ARE: Antioxidant response element
Csn1s1: Casein alpha s1
Csn1s2: Casein alpha s2
Csn2: Casein beta
Csn3: Casein kappa
DEG: Differentially expressed gene
ER: Endoplasmic reticulum
GCL: Glutamate cysteine ligase
LPS: Lipopolysaccharide
GSH: Glutathione
GSSG: Oxidized glutathione
Nrf2: Nuclear factor E2-related factor 2
PCR: Polymerase chain reaction
T-AOC: Total antioxidant capacity
WT: Wild-type.

## Supplementary Materials

Supplementary Materials Supplemental Figure 1: the genotype identification and reproductive performance of mice. Supplemental Figure 2: overview of RNA-seq in the mammary gland of mice. Supplemental Figure 3: comparison of the mRNA fold change of DEGs in the mammary gland by qPCR and RNA-Seq analysis. Supplemental Figure 4: GO and KEGG enrichment analysis of DEGs between the mammary glands treated with LPS or PBS in WT and Nrf2(-/-) mice, respectively. Supplemental Figure 5: protein-protein interaction (PPI) network analysis in WT and Nrf2(-/-) mice. Supplemental Table 1: sequences of primers used in real-time PCR.
