# Supplementary material for: Nrf2-ARE Signaling Partially Attenuates Lipopolysaccharide-Induced Mammary Lesions via Regulation of Oxidative and Organelle Stresses but Not Inflammatory Response in Mice
Source: Oxid Med Cell Longev. 2021 Jan 8;2021:8821833. doi: 10.1155/2021/8821833 (PMC7810562; doi:10.1155/2021/8821833)
Supplement: Supplementary Materials — Supplemental Figure 1: the genotype identification and reproductive performance of mice. Supplemental Figure 2: overview of RNA-seq in the mammary gland of mice. Supplemental Figure 3: comparison of the mRNA fold change of DEGs in the mammary gland by qPCR and RNA-Seq analysis. Supplemental Figure 4: GO and KEGG enrichment analysis of DEGs between the mammary glands treated with LPS or PBS in WT and Nrf2(-/-) mice, respectively. Supplemental Figure 5: protein-protein interaction (PPI) network analysis in WT and Nrf2(-/-) mice. Supplemental Table 1: sequences of primers used in real-time PCR. [file 8821833.f1.zip › Revised Supplemental material.docx]

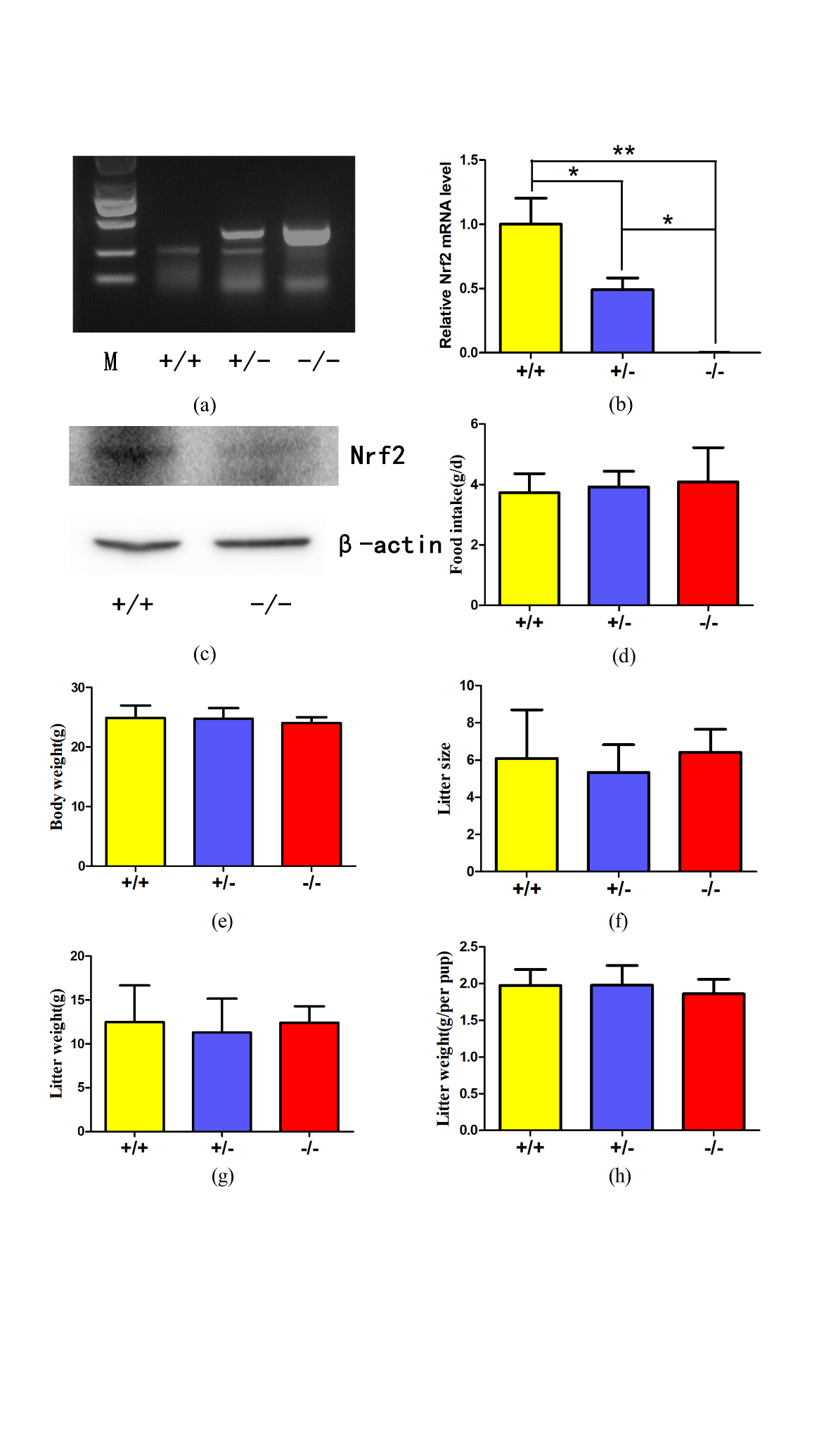


**Supplemental Figure 1**. (a)The [genotype](javascript:;) [identification](javascript:;) of mice (n=3), (b) relative mRNA abundance of Nrf2 (n=3) and (c) protein abundance of Nrf2 (n=3) in wild-type (WT), Nrf2(+/-) and Nrf2(-/-) mice. Effects of Nrf2 on (d) food intake (n=6), (e) body weight (n=12), (f) litter size (n=12), (g) litter weight (n=12) and (h) litter weight per pup (n=12) in WT, Nrf2(+/-) and Nrf2(-/-) mice. ANOVA followed by Tukey’s multiple comparison were used to determine the differences among three genotypes of mice. Data represent the mean ± SEM.


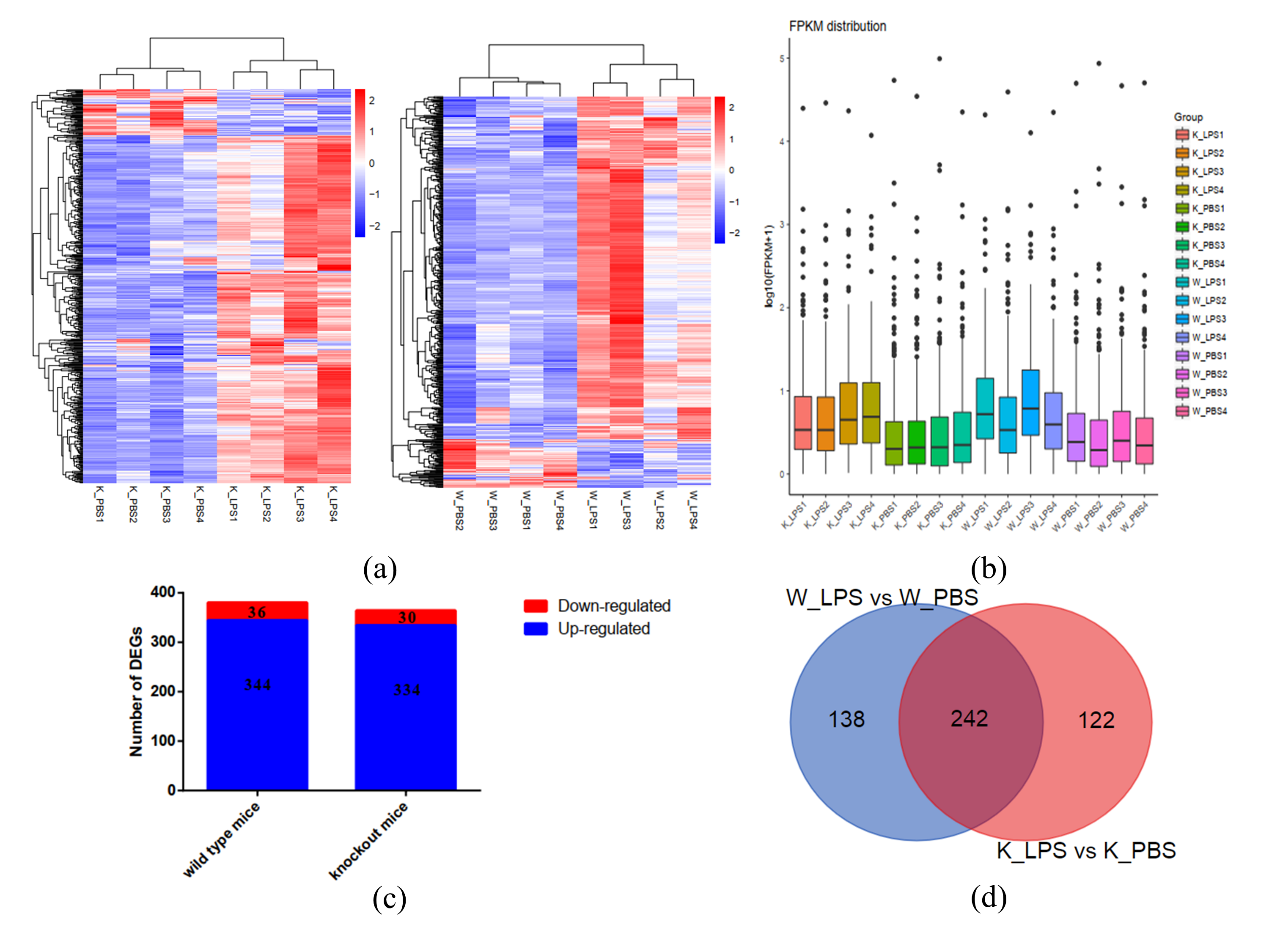


**Supplemental Figure 2.** Overview of RNA-seq in the mammary gland of wild type (W) and Nrf2(-/-) (K) mice treated with either LPS or PBS (n=4). (a) The heat maps for differentially expressed genes (DEGs) between LPS and PBS treated groups. The color code indicates upregulated (blue) and downregulated (red) expression levels. (b) Gene expression distribution in WT and Nrf2(-/-) mice. (c) Histogram indicates the number of DEGs (not including novel genes) between LPS and PBS treated groups in WT and Nrf2(-/-) mice. (d) Venn diagrams of the DEGs (Padj <0.01, fold change ≥2) between LPS and PBS treated groups in WT and Nrf2(-/-) mice.


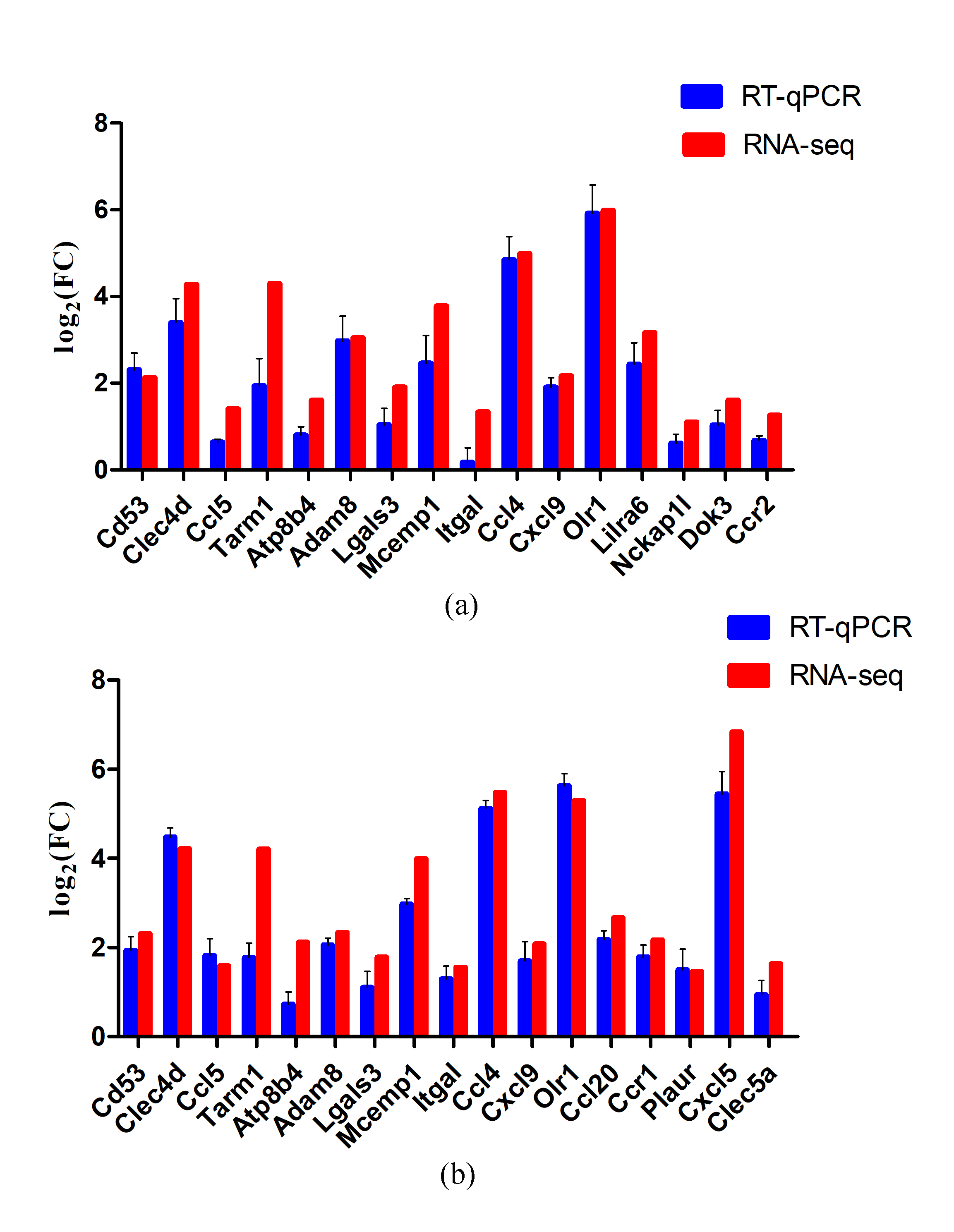


**Supplemental Figure 3.** (a) Comparison of the mRNA fold change of selected DEGs in WT mice by qPCR and RNA-Seq (n=4), and (b) Comparison of the mRNA fold change of selected DEGs in Nrf2(-/-) mice by qPCR and RNA-Seq (n=4).


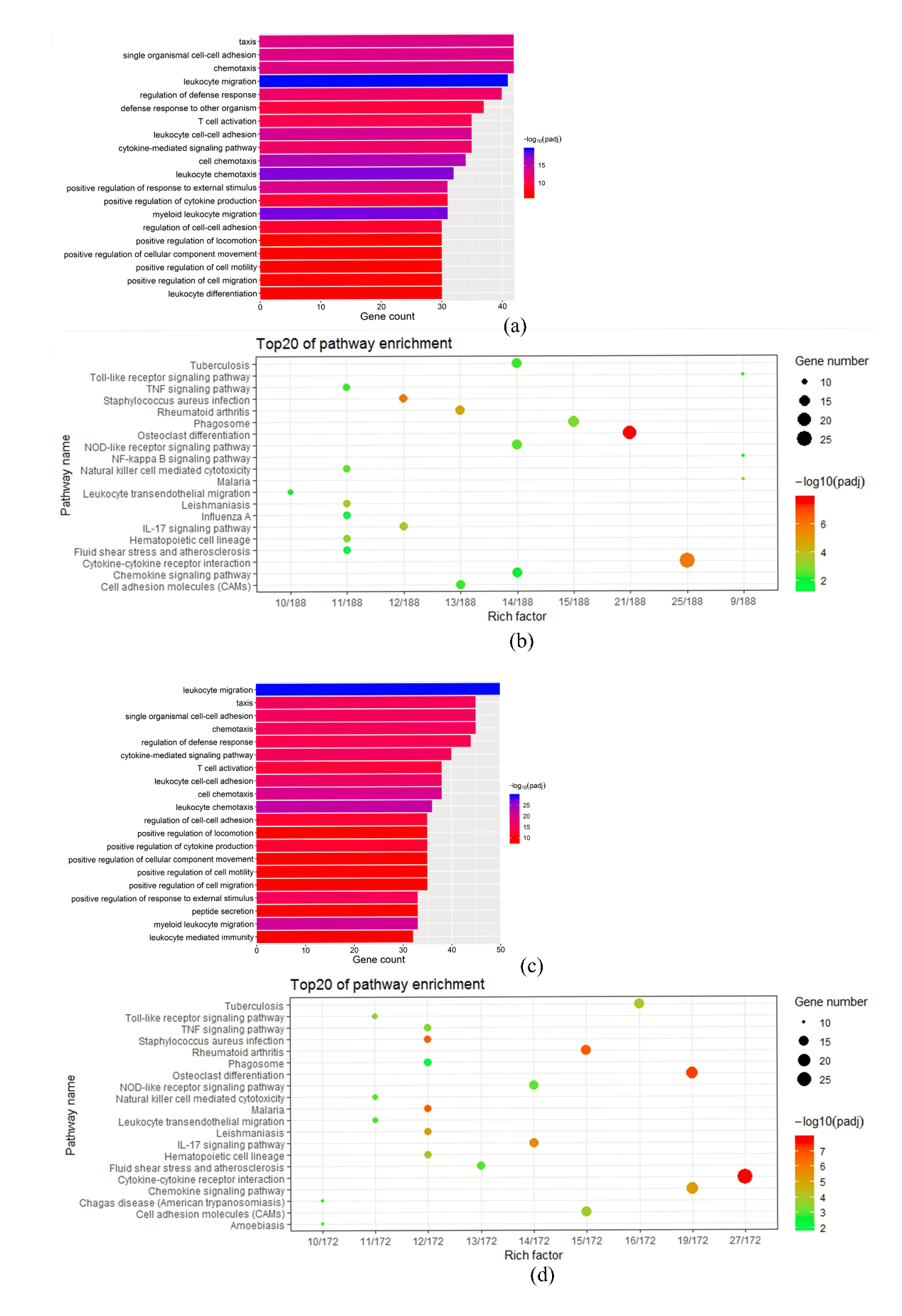
**Supplemental Figure 4.** Gene ontology (GO) and Kyoto Encyclopedia of Genes and Genomes (KEGG) enrichment analysis of differentially expressed genes (DEGs) between the mammary glands treated with LPS or PBS in WT and Nrf2(-/-) mice [respectively](javascript:;) (n=4). (a) GO categories assigned to the DEGs between LPS and PBS treated groups in the WT mice. (b) KEGG categories assigned to the DEGs between LPS and PBS treated groups in the WT mice. (c) GO categories assigned to the DEGs between LPS and PBS treated groups in the Nrf2(-/-) mice. (d) KEGG categories assigned to the DEGs between LPS and PBS treated groups in the Nrf2(-/-) mice. The top 20 biological process terms and KEGG terms are shown (Padj <0.05).


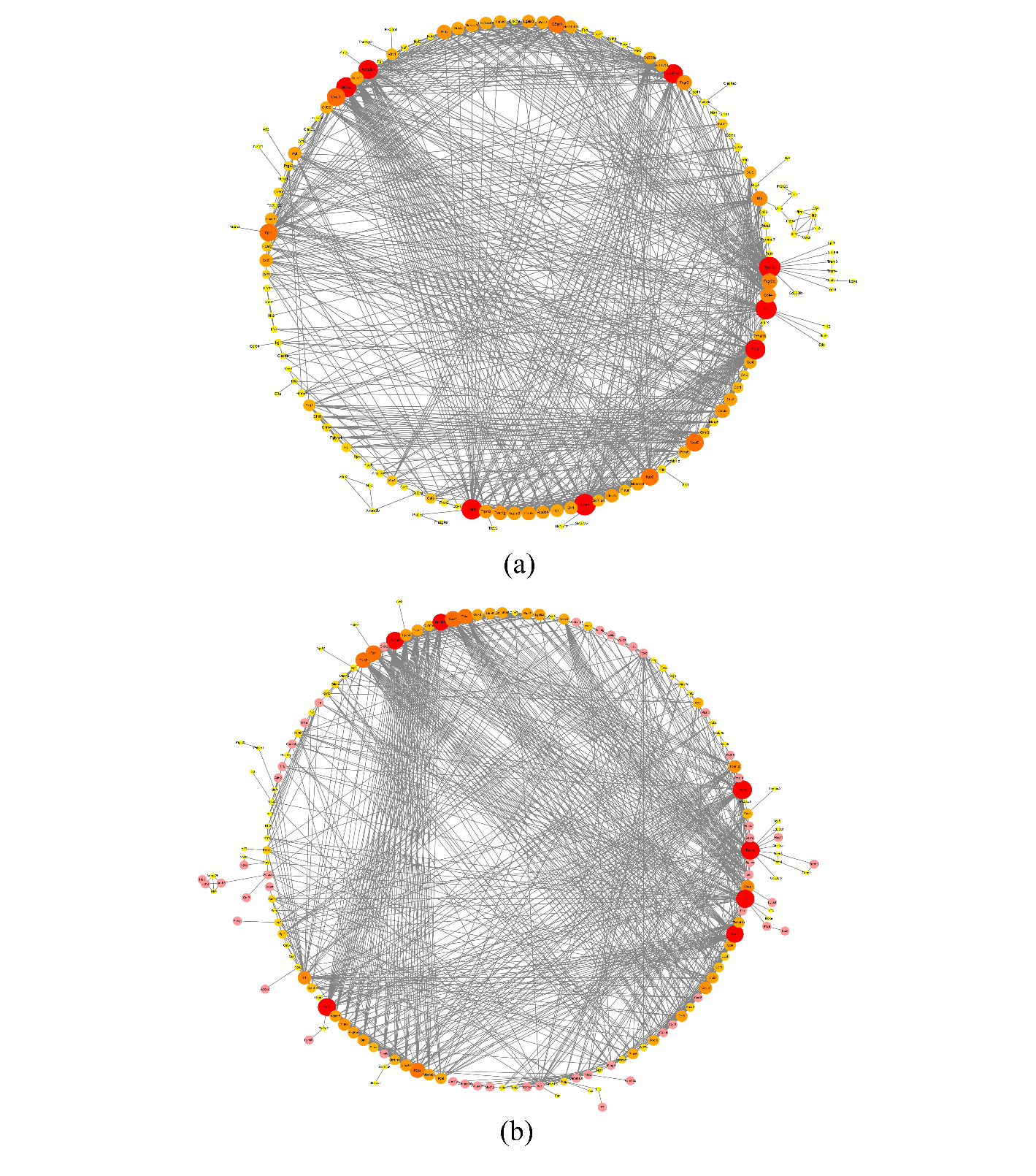


**Supplemental Figure 5.** Protein-protein interaction (PPI) network analysis in WT and Nrf2(-/-) mice, respectively (n=4). (a) PPI network of DEGs in WT mice between LPS and PBS treatment (b) PPI network of DEGs in Nrf2(-/-) mice between LPS and PBS treatment.

**Supplemental Table 1.** Sequences of primers used in real-time PCR

| Gene | Forward (5’-3’) | Reverse (5’-3’) | GenBank Accession |
| --- | --- | --- | --- |
| Arbp | TTCGTGTTCACCAAGGAG | TGGAGATTTTCGTGGTGATG | [NM_007475.5](https://www.ncbi.nlm.nih.gov/entrez/viewer.fcgi?db=nucleotide&id=254939638) |
| Sdha | CTGGCTGCCTCATCACAGAA | AATTCCGGGTAGACGTGTGG | [NM_023281.1](https://www.ncbi.nlm.nih.gov/entrez/viewer.fcgi?db=nucleotide&id=54607097) |
| Hprt1 | GCAGTCCCAGCGTCGT | GGCCTCCCATCTCCTTCAT | [NM_013556.2](https://www.ncbi.nlm.nih.gov/entrez/viewer.fcgi?db=nucleotide&id=96975137) |
| β-actin | GGCTGTATTCCCCTCCATCG | CCAGTTGGTAACAATGCCATGT | [NM_007393.5](https://www.ncbi.nlm.nih.gov/entrez/viewer.fcgi?db=nucleotide&id=930945786) |
| B2m | GAAGCCGAACATACTGAACTG | CACATGTCTCGATCCCAGTAG | [NM_009735.3](https://www.ncbi.nlm.nih.gov/entrez/viewer.fcgi?db=nucleotide&id=144227219) |
| Gapdh | CGTGCCGCCTGGAGAAACCTG | AGAGTGGGAGTTGCTGTTGAAGTCG | [NM_001289726.1](https://www.ncbi.nlm.nih.gov/entrez/viewer.fcgi?db=nucleotide&id=576080554) |
| Gclc | GATGATGCCAACGAGTCTGA | GACAGCGGAATGAGGAAGTC | [NM_010295.2](https://www.ncbi.nlm.nih.gov/entrez/viewer.fcgi?db=nucleotide&id=324710985) |
| Gclm | GGAGGGGCTCTTAACTCCAG | CTCAACACAGTGCCGAACAA | NM_008129.4 |
| IL-1β | TGCCACCTTTTGACAGTGATG | AAGGTCCACGGGAAAGACAC | NM_008361.4 |
| Ccl3 | CAGCGAGTACCAGTCCCTTT | GCAGTGGTGGAGACCTTCAT | NM_011337.2 |
| Cxcr2 | ATAGTGTTGACTGGAGGCTGG | TCCCTAACTGGAGCTGTGTCT | NM_009909.3 |
| Nrf2 | CAGCATGTTACGTGATGAGG | GCTCAGAAAAGGCTCCATCC | [NM_010902.4](https://www.ncbi.nlm.nih.gov/entrez/viewer.fcgi?db=nucleotide&id=927028865) |
| Nqo1 | TTCTCTGGCCGATTCAGAGTG | GTGGCCAATGCTGTAAACCAG | [NM_008706.5](https://www.ncbi.nlm.nih.gov/entrez/viewer.fcgi?db=nucleotide&id=161621259) |
| xCT | AGCCAGTCGGTGATAGCAAAG | AGGGGGAAAAACAAAACAAGAC | NM_011990.2 |
| Ho-1  CAT  BAX | ACATTGAGCTGTTTGAGGAG  CTTTCCCATTTAATCCATTTG  AGCAAACTGGTGCTCAAGGC | TACATGGCATAAATTCCCACTG  GGCCCTGAAGCATTTTGTC  CCACAAAGATGGTCACTGTA | NM_010442.2  NM_009804.2  [NM_007527.3](https://www.ncbi.nlm.nih.gov/entrez/viewer.fcgi?db=nucleotide&id=133778943) |
| Bcl-xl | GTATTGGTGAGTCGGATTGC | TGGACGGTCAGTGTCTGG | [NM_001355053.1](https://www.ncbi.nlm.nih.gov/entrez/viewer.fcgi?db=nucleotide&id=1243938591) |
| Csn1s1 | GCAACAGCATAGCAGCAGTG | GCTGCATCCATAGTTACCTTGATT | NM_007784.3 |
| Csn1s2 | CCATTGCCTGGACTACTTGTCT | TTCTTTGCAAGGGCAACAGC | NM_174528.2 |
| Csn2 | CTCTCTTGTCCTCCACTAAAGGTA | AGGCTGGATGTTTTGTGGGA | NM_001286021.1 |
| Csn3 | TGCCATTCCAAACCCATCCT | ATGATGGCAGAGTTTCCCCTG | NM_001356570.1 |
| Chop | GGAACCTGAGGAGAGAGTGTT | GACTGGAATCTGGAGAGCGA | NM_007837.4 |
| Gpr78 | TCGATACTGGCCGAGACAAC | GGAGACACGAAGCAGACTCG | NM_022310.3 |
| Cd53 | CCGTAACCTTCCCTTCCTGAC | TTGATTGAGCCCATGCAACC | [NM_007651.3](https://www.ncbi.nlm.nih.gov/entrez/viewer.fcgi?db=nucleotide&id=161484612) |
| Cxcl5 | TGTCCACAATGAGCCTCCAG | GCTATGACTGAGGAAGGGGC | NM_009141.3 |
| Clec5a | ACATTACCGAGCAGGAGCAT | TGCACACTAGTGGTTCCGTAG | NM_001038604.1 |
| Ccl20 | CAGGCAGAAGCAGCAAGCAAC | CCCCAGCTGTGATCATTTCCT | NM_016960.2 |
| Plaur | GACCCACCTCAACGTCTCTG | AGTCAGGTCCAGAGGAGGAC | NM_011113.4 |
| Ccr1 | ACCTGTTCAACCTGGCTGTC | AAGCTTGCACATGGCATCAC | NM_009912.4 |
| Ccr2 | GCCATCATAAAGGAGCCATACC | ATGCCGTGGATGAACTGAGG | NM_009915.2 |
| Clec4d | GGACGAGAGGAAGTGTGGTG | TCATGCCAGGTCTGGTTGTC | NM_010819.4 |
| Ccl5 | GCTCCAATCTTGCAGTCGTG | GAGCAGCTGAGATGCCCATT | NM_013653.3 |
| Tarm1 | TGCGTTACTGAAGGTGGGAC | CAGCTGTATTCCCCCGAGTC | NM_177363.4 |
| Atp8b4 | CTTTTGCGTGGGTGTGCTTAC | TTCACCACGTCATGGGTGTC | NM_001359975 |
| Olr1 | CCCTGCTGCTATGACTCTGG | GCTGAGTAAGGTTCGCTTGG | NM_138648.2 |
| Adam8 | ATCCCATCATGCTTGGCCTC | AAAGGAGGTCCAGGGGCTAC | NM_007403 |
| Lgals3 | TAATCAGGTGAGCGGCACAG | GTAGGCCCCAGGATAAGCAG | NM_001145953.1 |
| Mcemp1 | GCCTCCCAGGATAAGAACCG | CTCCCGGATATTCCACACCG | NM_026985.1 |
| Itgal | CCATGCAGCCTATCCTGAGAC | GTGGCTGAAGCATCTCCACT | NM_001253872.1 |
| Nckap1l | AGGAATATCCAGGGCTGAGG | TCTCTGCCCGCCAACATTAT | NM_153505.4 |
| Lilra6 | GTGTGGGGTTCAGAGGGAAG | GCAGGTGGGACAGCCTATTC | NM_011090.2 |
| Dok3 | GACCAATGGGGGTCCTGAAG | TTCTTCCGTTCACGGGTCAG | NM_013739.2 |
| Ccl4 | CCAGGGTTCTCAGCACCAAT | TTGGAGCAAAGACTGCTGGT | NM_013652.2 |
| Cxcl9 | AACGTTGTCCACCTCCCTTC | CACAGGCTTTGGCTAGTCGT | NM_008599.4 |
