# Supplementary figures and images for: Nrf2-ARE Signaling Partially Attenuates Lipopolysaccharide-Induced Mammary Lesions via Regulation of Oxidative and Organelle Stresses but Not Inflammatory Response in Mice
Source: Oxid Med Cell Longev. 2021 Jan 8;2021:8821833. doi: 10.1155/2021/8821833 (PMC7810562; doi:10.1155/2021/8821833)

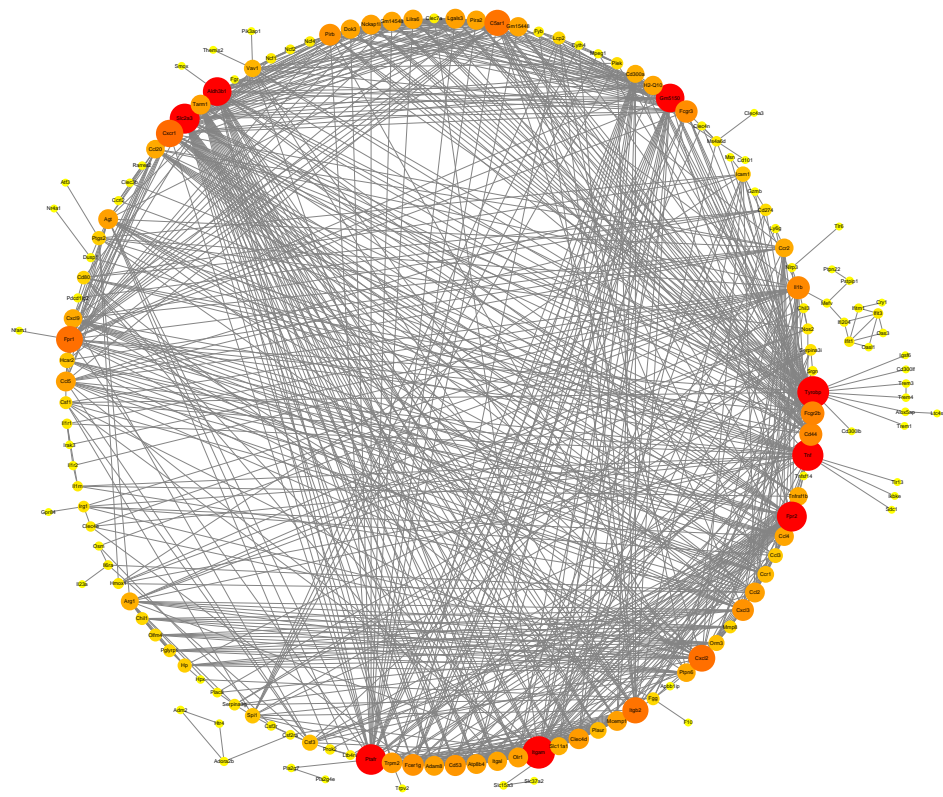

Supplement: Supplementary Materials — Supplemental Figure 1: the genotype identification and reproductive performance of mice. Supplemental Figure 2: overview of RNA-seq in the mammary gland of mice. Supplemental Figure 3: comparison of the mRNA fold change of DEGs in the mammary gland by qPCR and RNA-Seq analysis. Supplemental Figure 4: GO and KEGG enrichment analysis of DEGs between the mammary glands treated with LPS or PBS in WT and Nrf2(-/-) mice, respectively. Supplemental Figure 5: protein-protein interaction (PPI) network analysis in WT and Nrf2(-/-) mice. Supplemental Table 1: sequences of primers used in real-time PCR. [file 8821833.f1.zip › Supplemental figure 5(a).pdf]

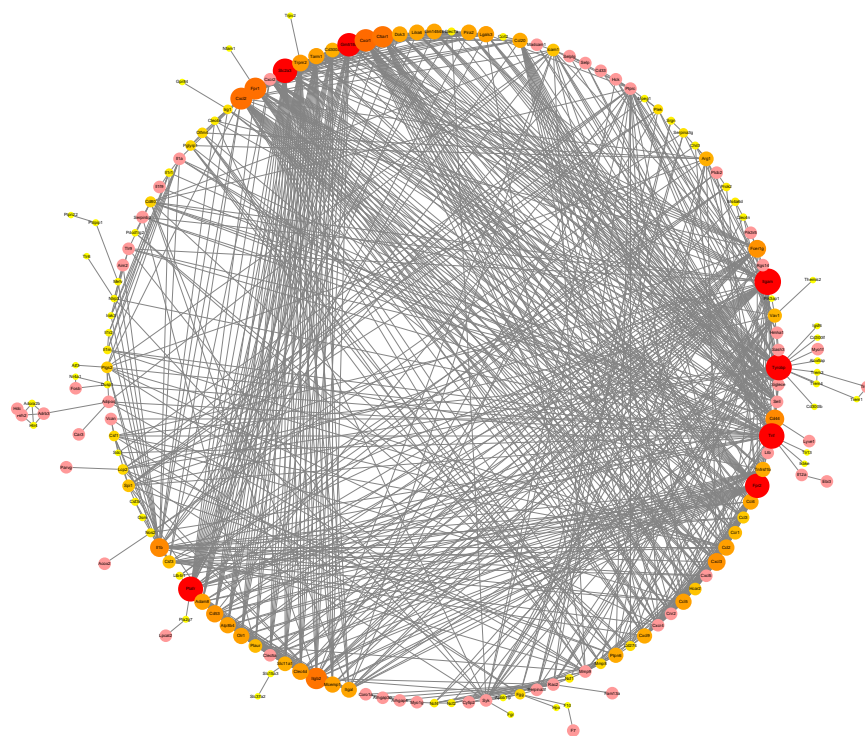

Supplement: Supplementary Materials — Supplemental Figure 1: the genotype identification and reproductive performance of mice. Supplemental Figure 2: overview of RNA-seq in the mammary gland of mice. Supplemental Figure 3: comparison of the mRNA fold change of DEGs in the mammary gland by qPCR and RNA-Seq analysis. Supplemental Figure 4: GO and KEGG enrichment analysis of DEGs between the mammary glands treated with LPS or PBS in WT and Nrf2(-/-) mice, respectively. Supplemental Figure 5: protein-protein interaction (PPI) network analysis in WT and Nrf2(-/-) mice. Supplemental Table 1: sequences of primers used in real-time PCR. [file 8821833.f1.zip › Supplemental figure 5(b).pdf]
